# Polysomnography and Nocturia Evaluations after Uvulopalatopharyngoplasty for Obstructive Sleep Apnea Syndrome

**DOI:** 10.3390/jcm9103089

**Published:** 2020-09-25

**Authors:** Yung-An Tsou, Eric Chieh-Lung Chou, Dung-Yun Shie, Ming-Jeng Lee, Wen-Dien Chang

**Affiliations:** 1 Department of Otolaryngology-Head and Neck Surgery, China Medical University Hospital, Taichung 40402, Taiwan; d22052121@gmail.com (Y.-A.T.); adongwin@yahoo.com.tw (D.-Y.S.); 2 Department of Audiology and Speech-Language Pathology, Asia University, Taichung 40402, Taiwan; 3 School of Medicine, China Medical University, Taichung 40402, Taiwan; ericchou66@yahoo.com.tw; 4 Department of Genitourology, China Medical University Hospital, Taichung 40402, Taiwan; 5 Department of Bioengineering, Rice University, Houston, TX 77005, USA; MingJeng.Rose.Lee@gmail.com; 6 Department of Sport Performance, National Taiwan University of Sport, Taichung 40404, Taiwan

**Keywords:** obstructive sleep apnea, uvulopalatopharyngoplasty, nocturia

## Abstract

A higher incidence rate of nocturia in patients with obstructive sleep apnea (OSA) has been observed. We investigated the differences in clinical examinations between OSA patients with and without nocturia, and further compared those with successful and unsuccessful uvulopalatopharyngoplasty (UPPP). This retrospective study enrolled 103 patients with OSA undergoing UPPP. Patients were diagnosed with OSA by following the 2018 American Academy of Sleep Medicine (AASM) Scoring Manual Version 2.5. Patients were divided into two groups depending on if they urinated more than twice per night. The medical data of body mass index (BMI), nocturia frequency per night, apnea–hypopnea index (AHI), Epworth Sleepiness Scale (ESS), International Prostatic Symptom Score (IPSS), and Overactive Bladder Symptom Score (OABSS) were analyzed before and after uvulopalatopharyngoplasty (UPPP) surgery. All of the measurements were compared between successful and unsuccessful surgery in the non-nocturia or nocturia groups, respectively. Fifty patients (41 males and nine females) without nocturia were assigned to group 1, and 53 patients (43 males and 10 females) with nocturia were assigned to group 2. Nocturia frequency and post-surgery AHI in group 2 were significantly higher than those in group 1 (*p* < 0.05). Significant decreases in IPSS and OABSS were observed in the successful surgery subgroup of group 2 (*p* < 0.05). A significant decrease in post-surgery AHI was observed between unsuccessful and successful surgery in patients with nocturia (*p* < 0.05), but not in the non-nocturia group (*p* > 0.05). Although AHI had a significant correlation to nocturia frequency in all OSA patients before UPPP, no significant correlation between AHI reduction and nocturia frequency was found. UPPP appeared to be an effective treatment for nocturia associated with OSA. OSA should be taken into consideration for patients who complain of nocturia syndrome. The relationship of AHI reduction and nocturia improvement after OSA treatment with UPPP is still unclear. In addition, it is necessary to establish the existence of nocturia in patients with OSA, as a result of its high prevalence in OSA patients. UPPP could reduce the symptoms of OSA and could also contribute to a reduction of nocturia even in the unsuccessful surgery group.

## 1. Introduction

Patients with obstructive sleep apnea (OSA) have daytime symptoms such as excessive daytime sleepiness, poor concentration, and agitation in social relationships [1]. Poor working efficacy also leads to low-quality performance during work [2]. Some OSA patients have even experienced traffic and work safety issues [3]. Nighttime symptoms include snoring, sleep apnea, desaturation during sleep, and frequent arousals [4,5]. Frequent arousal may be caused by desaturation, leading to changes in the sleep stage, i.e., from deep to shallow sleep.

There is a higher incidence rate of nocturia in patients with OSA [6]. Nocturia is also considered a multi-etiology symptom during nighttime sleep, but rather for any condition affecting stable sleep architecture [7,8]. Reports and meta-analyses have revealed the success of treating sleep apnea using continuous positive airway pressure (CPAP) ventilation in order to reduce the frequency of nocturia by means of questionnaire surveillance [9]. In addition, it has been reported that sleep surgery, i.e., uvulopalatopharyngoplasty (UPPP), can successfully reduce the frequency of nocturia [10]. However, it is difficult to determine the effect of UPPP on improvement of nocturia, due to the lack of non-nocturia OSA patients as the compared group, and small sample size (*n* = 37) [10]. Comparing the clinical effects between nocturia and non-nocturia OSA patients could clarify the effects of UPPP on nocturia.

Nowadays, it is thought that the low arousal threshold leading to changes in sleep is not only caused by desaturation, but also by increased sensory sensations regarding urinary urgency and by intolerance to urinary input sensations, even when there is no urine in the bladder, causing increased bladder wall pressure [11]. The association between the severity of sleep apnea and the frequency of nocturia is still being studied. Patients with severe OSA and a higher apnea–hypopnea index (AHI) have been reported to experience a higher frequency of nocturia [7,12]. Successful treatment of OSA leads to deeper sleep, but also to increased arousal thresholds during sleep [13]. Some studies have indicated that conservative treatments, such as CPAP, could improve nighttime urination frequency and decrease the volume of nocturnal urine [14,15]. Little research has investigated the possibility that successful surgery for OSA could improve nocturia. Therefore, we hypothesized that successful UPPP for OSA may cause a decreased frequency of nocturia. The aim of our study was to compare the differences in clinical examinations between OSA patients with and without nocturia and, furthermore, to compare those between successful and unsuccessful surgery subgroups.

## 2. Methods

This was a retrospective study. All patients’ data were protected under the Internal Review Board of China Medical University Hospital (CMUH106-REC2-027). All routine care and treatments were offered according to clinical guidelines. The sample size was determined a priori using measures of effect size and based on a previous study for nocturia following surgery in OSA patients [10]. Power analysis revealed a necessary sample size of 37, as well as an Overactive Bladder Symptom Score (OABSS) that significantly decreased from 2.9 ± 2.2 to 2.0 ± 2.2 after surgery (*p* = 0.005), as found in the study of Park et al. [10]. It was also determined that a power of 0.8 and an alpha of 0.05, with at least a total of 45 participants, would be required to represent a sufficient quantity.

The charts of 103 OSA patients undergoing UPPP were enrolled for review during the course of this study. Patients eligible for UPPP were diagnosed with OSA with an AHI >5/h by using polysomnography following the guidance of the 2018 American Academy of Sleep Medicine (AASM) Scoring Manual Version 2.5 at China Medical University Hospital [16], Taichung, Taiwan, between 2016 and 2019. The inclusion criteria were that the OSA patients had already planned to undergo UPPP due to poor efficacy of CPAP. Patient selection for UPPP surgery is dependent upon physical examination, revealing at least grade II enlarged palatine tonsils by the Friedman staging system, associated with elongated uvula, pillar webbing, and a redundant soft palate. The exclusion criteria were male patients with benign prostate hypertrophy (BPH) or with a BPH operation medical history by two genitourinary doctors and an otolaryngologist. Patients with hypertension, diabetes, renal disorders, or other major medical diseases such as heart failure or chronic lung diseases were also excluded. The question “Over the past month, how many times did you most typically get up to urinate from the time you went to bed at night until the time you got up in the morning?” [17] was used to assess the number of nocturia episodes. All patients were under a fluid restriction of 100 mL three hours before the study period. The definition of night nocturia was if the patient had equal to or more than two urinations per night [18]. We used this standard to classify patients into two groups, namely, OSA patients without nocturia (group 1) and OSA patients suffering from night nocturia (group 2). To go a step further, OSA patients suffering from nocturia were later divided into two subgroups after the treatment based on the surgical success criteria, which were the reduction of at least 50% in preoperative AHI or a postoperative AHI of less than 20 per hour [19].

### 2.1. Polysomnography

All patients were assessed for OSA and AHI before UPPP surgery, and we prescribed post-UPPP polysomnography (PSG) as well as PSG 6 months after surgery. All of the outpatients received a prescription of PSG 2 months after operation but all of them received a post-op PSG follow-up in the 6 month period after surgery (because the waiting list in our center is 4 months at least), therefore, all of our patients received PSG 6 months after surgery. The Embla N7000 system (Medcare-Embla, Reykjavik, Iceland) was used to measure brain waves, sleep architecture, oxygen saturation, heart rate, respiratory movement, and periodic leg movements (PLMs) during sleep at night. The polysomnography data were collected and AHI was assessed by one physician. The AHI is defined as total apnea and hypopnea per hour during a night of sleep. Apnea was defined as airflow <10% of the average flow for more than 10 s with 4% O_2_ desaturation or stoppage of airflow [20]. Obstructive hypopnea was defined as >30% decreased airflow from the average baseline breath for more than 10 s with 4% O_2_ desaturation [20].

### 2.2. Uvulopalatopharyngoplasty

The surgical operation was performed by three otolaryngologists. The redundant posterior pillar tissue and tonsils were shaved and removed, and then the anterior and posterior pillars were sutured. All surgeries were performed at the China Medical University Hospital.

### 2.3. Clinical Assessments

After conducting basic medical examinations of the patients and receiving the research-related indices, such as body mass index (BMI), nocturia frequency, AHI, Epworth Sleepiness Scale (ESS), International Prostatic Symptom Score (IPSS), and Overactive Bladder Symptom Score (OABSS), we performed UPPP on all patients. The BMI, AHI, ESS, IPSS, and OABSS were assessed 6 months post-surgery by the original otolaryngologist, and then these clinical assessments were compared between pre- and post-UPPP in OSA patients with and without nocturia.

#### 2.3.1. Epworth Sleepiness Scale

The ESS is a self-administered questionnaire to assess symptoms associated with falling asleep in eight different situations. Each item of the ESS was scored from 0 to 3, and the total scores ranged from 0 to 24. A higher ESS score means that patients have higher sleep propensity in daily life [21].

#### 2.3.2. International Prostatic Symptom Score

The IPSS is determined by a seven-item self-administered questionnaire, which contains questions concerning urinary symptoms. A higher IPSS score indicates an increased severity of urinary symptoms. Each IPSS question was calculated, and the total scores ranged from 0 to 35 [22].

#### 2.3.3. Overactive Bladder Symptom Score

The OABSS is a reliable and valid assessment tool for evaluating fluctuations in overactive bladder symptoms. Four items in the OABSS questionnaire, including daytime frequency, nighttime frequency, urgency, and urge incontinence, were weighted to assess the frequency of urgency and urgency incontinence. The maximum score of OABSS is 15 [23].

### 2.4. Statistical Analysis

This research applied parameter analysis for the comparison of nocturia frequency and the AHI to evaluate the severity of OSA. In addition, the changes in nocturia frequency before and after UPPP were taken into account in the parameter analysis as well. All analyses were performed using SPSS 19 (SPSS Inc., Chicago, IL, USA). We used the Shapiro–Wilk test to verify the normality of the measured data, and found that they were normally distributed (*p* > 0.05). To compare patients’ characteristics in this study, the *t*-test and the chi-squared test were used for analysis. For the comparisons of IPSS and OABSS, the *t*-test and the paired *t*-test were used for analysis, which are represented in the figures. The Mann–Whitney U test was used to compare the changes of AHI and nocturia frequency after UPPP among different OSA severity groups, which were classified as mild (AHI = 5~14/h), moderate (AHI = 15~30/h), and severe (AHI > 30/h). The Spearman test was used further to analyze the correlation between OSA reduction and nocturia effects. BMI, nocturia frequency, AHI, and ESS data were analyzed using a two-way repeated measures analysis of variance (ANOVA) (group or subgroup × time), followed by the Bonferroni post hoc test. The main effects of time represented the changes in the clinical assessment measure pre- and post-surgery. A *p*-value < 0.05 was considered statistically significant, and data from all measures are presented as mean ± standard deviation.

## 3. Results

In this study, a total of 103 (84 males and 19 females) OSA-diagnosed patients were enrolled. These patients were classified into two groups based on whether they suffered from a night nocturia frequency of more than two episodes per night. After the brief survey, we assigned 50 patients (41 males and nine females) without night nocturia to group 1 and 53 patients (43 males and 10 females) with night nocturia to group 2. There were no significant differences in terms of age, BMI, or ESS index (Table 1). We divided them into the two groups on the basis of equal or more than two episodes of nocturia per night. There was a significant difference in the AHI between group 1 and group 2 (*p* < 0.05). There were also significant differences in the AHI, IPSS, and OABSS, as well as in the number of night urinations in the comparison between groups 1 and 2 (*p* < 0.05; Table 1). As shown in Table 2, the changes in AHI and nocturia frequency in moderate (AHI = 15~30/h) and severe (AHI > 30/h) OSA were significantly lower than for mild (AHI = 5~14/h) OSA (*p* < 0.05).

**Figure 1:**
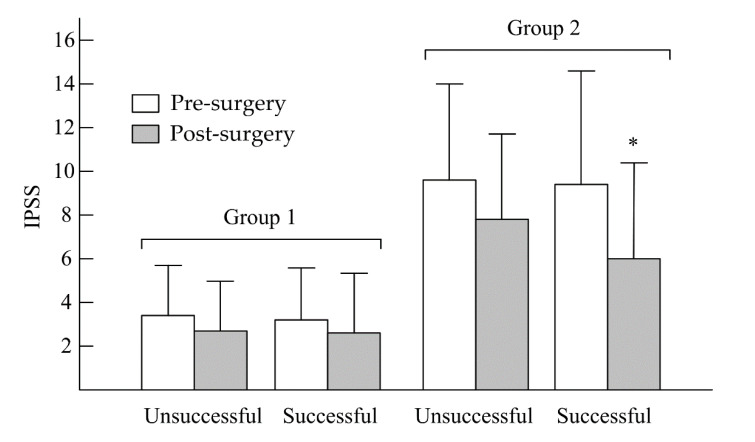
Changes in IPSS between pre- and post-uvulopalatopharyngoplasty (UPPP) in non-nocturia (group 1) and nocturia (group 2) patients. * *p* < 0.05, pre- vs. post-UPPP.

**Figure 2:**
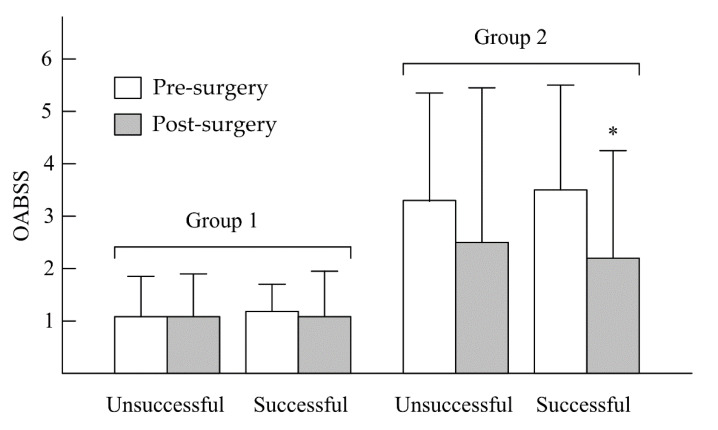
Changes in OABSS between pre- and post-UPPP in non-nocturia (group 1) and nocturia (group 2) patients. * *p* < 0.05, pre- vs. post-UPPP.

In Figure 1, after UPPP, the unsuccessful and successful surgery subgroups in group 1 showed no significant decreases in IPSS—from 3.42 ± 2.12 to 2.46 ± 2.47 (*p* > 0.05) and from 3.27 ± 3.01 to 2.68 ± 2.89 (*p* > 0.05), respectively. In group 2, no significant decreases in IPSS were found in the unsuccessful surgery subgroup (from 9.68 ± 5.33 to 7.79 ± 4.89, *p* > 0.05), but a significant decrease in IPSS was observed in the successful surgery subgroup (from 9.42 ± 6.76 to 6.46 ± 5.01, *p* < 0.05). In the unsuccessful and successful surgery subgroups of group 1 (Figure 2), there were no significant decreases in OABSS—from 1.01 ± 0.77 to 1.00 ± 0.82 (*p* > 0.05) and from 1.14 ± 0.62 to 1.01 ± 0.92 (*p* > 0.05), respectively. In group 2, the change in OABSS also did not show a significant decrease in the unsuccessful surgery subgroup (from 3.34 ± 2.44 to 2.53 ± 3.12, *p* > 0.05). A significant decrease in OABSS was found in the successful surgery subgroup (from 3.52 ± 2.33 to 2.12 ± 2.11, *p* < 0.05). Before undergoing UPPP, AHI had a significant correlation to nocturia frequency among all patients with OSA (r = 0.37, *p* = 0.001). However, we found that there were no significant correlations between AHI reduction and nocturia frequency after unsuccessful and successful surgery among non-nocturia or nocturia patients (*p* > 0.05). In non-nocturia patients undergoing unsuccessful and successful surgery, the correlations between AHI reduction and decrease of IPSS or OABSS were not found (*p* > 0.05). The AHI reduction in nocturia patients undergoing unsuccessful surgery also did not correlate to IPSS and OABSS (*p* > 0.05), but the nocturia patients undergoing successful surgery had a slight significant correlation with IPSS (r = 0.21, *p* = 0.04) or OABSS (r = 0.11, *p* = 0.03).

The changes in BMI, nocturia frequency, AHI, and ESS in groups 1 and 2 pre- and post-surgery are shown in Table 3. For BMI, we found main effects of time (F = 38.21, *p* = 0.001), group (F = 0.11, *p* = 0.82), and time × group (F = 0.49, *p* = 0.48). There was a significant main effect on BMI of time (*p* < 0.05). Post hoc tests showed that there were no significant differences within the group in BMI, neither in group 1 nor group 2. In group 1, BMI before the operation was 26.59, and after the operation, it was 26.89. In group 2, BMI before and after was 26.89 and 25.69, respectively. For nocturia frequency, main effects of time (F = 89.66, *p* = 0.001), group (F = 39.55, *p* = 0.01), and time × group (F = 72.99, *p* = 0.01) were noted. Significant main effects on nocturia frequency of time, group, and time × group were found (*p* < 0.05). For AHI, we noted main effects of time (F = 11.16, *p* = 0.002), group (F = 12.54, *p* = 0.001), and time × group (F = 14.72, *p* = 0.01). There were significant main effects on AHI of time, group, and time × group (*p* < 0.05). The number of urinations dropped noticeably from 2.27 to 0.84 per night and the AHI also decreased from 29.99 to 15.64 in group 2. The comparison of nocturia frequency between groups 1 and 2 was statistically significant (*p* = 0.001). Additionally, the results of nocturia frequency and the AHI pre- and post-surgery within group 2 were statistically significant (*p* = 0.001), but there was no such similarity in the results of group 1. For ESS, main effects of time (F = 2.15, *p* = 0.17), group (F = 3.95, *p* = 0.28), and time × group (F = 0.92, *p* = 0.32) were found. Post hoc tests showed that there was no statistically significant difference in ESS between the two groups.

Among non-nocturia patients, changes in BMI, nocturia frequency, AHI, and ESS pre- and post-surgery are shown in Table 4. For BMI, we found main effects of time (F = 1.91, *p* = 0.16), subgroup (F = 0.58, *p* = 0.45), and time × subgroup (F = 2.70, *p* = 0.11). The main effect on BMI of time was statistically significant (*p* < 0.05). BMI had a significant main effect of time (*p* < 0.05). For nocturia frequency, main effects of time (F = 1.49, *p* = 0.23), subgroup (F = 0.68, *p* = 0.79), and time × subgroup (F = 1.01, *p* = 0.33) were noted. No significant main effects on nocturia frequency of time, subgroup, or time × subgroup were noted (*p* > 0.05). For AHI, we found main effects of time (F = 0.05, *p* = 0.94), subgroup (F = 22.77, *p* = 0.001), and time × subgroup (F = 20.18, *p* = 0.001). There were significant main effects on AHI of subgroup and time × group (*p* < 0.05). Post hoc tests showed no significant differences in AHI pre- and post-surgery between the two subgroups (*p* > 0.05). For ESS, there were main effects of time (F = 2.26, *p* = 0.15), group (F = 0.43, *p* = 0.51), and time × group (F = 0.81, *p* = 0.38). No significant main effects on ESS of time, subgroup, and time × group were noted (*p* > 0.05). Post hoc tests showed no significant differences in BMI, nocturia frequency, AHI, or ESS between the two subgroups (*p* > 0.05).

Among nocturia patients, changes in BMI, nocturia frequency, AHI, and ESS pre- and post-surgery are shown in Table 5. For BMI, main effects of time (F = 30.54, *p* = 0.001), subgroup (F = 0.15, *p* = 0.86), and time × subgroup (F = 0.05, *p* = 0.94) were noted. For BMI, the only significant main effect was of time (*p* < 0.05). For nocturia frequency, main effects of time (F = 100.49, *p* = 0.001), subgroup (F = 0.27, *p* = 0.61), and time × group (F = 0.07, *p* = 0.78) were found. A significant main effect on nocturia frequency of time was noted (*p* < 0.05). In the successful surgery subgroup, there was an obvious improvement in the frequency of night urination, with nighttime urination scores of 2.39 for pre-surgery and 0.78 for post-surgery. For AHI, we noted main effects of time (F = 7.54, *p* = 0.01), group (F = 3.21, *p* = 0.04), and time × group (F = 16.48, *p* = 0.001). There were significant main effects on AHI of time, group, and time × group (*p* < 0.05). In the nocturia patients with successful surgery, post hoc tests showed a significant difference in the AHI in the successful surgery subgroup before and after the surgery of 34.81 ± 25.27 and 9.07 ± 11.41, respectively (*p* = 0.001). In the unsuccessful surgery subgroup, the AHI before and after surgery was 22.04 and 26.49, respectively. We noticed that in the unsuccessful surgery subgroup, even though the AHI was not improved, nocturia frequency decreased from 2.35 episodes per night before UPPP to 0.59 episodes per night afterward. For ESS, main effects of time (F = 4.24, *p* = 0.10), group (F = 0.31, *p* = 0.58), and time × group (F = 1.74, *p* = 0.19) were found. Post hoc tests showed that there were no significant differences in BMI, nocturia frequency, or ESS, but there was in the AHI between the two subgroups (i.e., successful or unsuccessful surgery).

## 4. Discussion

In our study, of the 53 nocturia patients, 33 patients met the criteria for successful surgery, and the other 20 patients were defined as the unsuccessful surgery subgroup. We conducted a comprehensive survey and recorded their physical information such as BMI, nocturia frequency, AHI, and ESS for comparison between the pre-surgical and post-surgical conditions. The successful definition was based on a reduction in AHI of over 50% and on having a reduction in AHI of less than 20 per hour according to Sher’s criteria [24]. It was evident that nocturia frequency greatly improved in both groups after the UPPP surgery, despite the fact that the AHI was not as high as expected in the unsuccessful surgery subgroup.

In the current study, we found that UPPP benefited nocturia patients in terms of decreasing the episodes of nocturia. A significant correction between AHI and nocturia frequency before UPPP was noted in the all of OSA patients (*p* < 0.05). However, we found no significant correlations between AHI reduction and nocturia frequency in unsuccessful and successful surgery in nocturia patients. Some studies supported that curing OSA could decrease nocturia episodes, and UPPP is an effective surgery for OSA [10,11]. Although a decrease in AHI was a criterion for successful UPPP on OSA, the correlation between AHI reduction and nocturia frequency was not found in our nocturia patients with OSA. This finding demonstrated that UPPP may have multifactorial factors affecting nocturia episodes. AHI improvement is only one factor that causes a decrease in nocturia, and the unclear mechanism still needs to be investigated. However, the AHI was observed to be well improved in the nocturia patients after surgery, which is compelling evidence for UPPP as a solution to enhance the life quality of nocturia patients, including OSA patients with nocturia syndrome. After UPPP, we carried out further experimental analysis by breaking down the nocturia patients into two subgroups on the basis of Sher’s success criteria, namely, those with more than a 50% reduction in AHI and those with a reduction in AHI of less than 20 per hour. A previous study showed that non-CPAP treatment, such as lifestyle modifications and behavioral treatments [25], pharmacotherapy [26], and surgical operations for bladder outlet obstruction relief, play an important role in reducing the frequency of nocturia [27]. There are few reports that discuss the relationship between UPPP and nocturia. Therefore, to the best of our understanding, this research is the largest clinical report focusing UPPP surgery as an efficient and powerful treatment for nocturia patients and as an effective solution to reduce AHI for patients.

Wang et al. indicated that successful treatments for OSA could provide a reduction in nighttime urine volume to improve nocturia [15]. Low reabsorbed percentages of filtered sodium and a higher urinary flow were shown in a previous study [28]. In clinical findings, the excretion of atrial natriuretic peptide has been found in OSA patients, who also had a higher respiratory disturbance index in their polysomnography results [25,29]. This symptom led to an increase in the total number of apnea events and hypopnea throughout the entire sleep period. A potential reason for the effects on nocturia is that successful treatments could reduce the excretion of atrial natriuretic peptide in OSA patients [27]. A systematic review and meta-analysis provided evidence of a decrease in the effects of polysomnography and symptoms as a result of UPPP in adult OSA patients, and found that this surgery is effective both in the short and long term post-surgery [30]. UPPP can effectively increase the lowest arterial oxygen saturation and decrease the percentage of sleep time with oxyhemoglobin saturation <90% in patients with OSA [30]. These effects could reduce the airway obstruction and excessive expansion of the cardiac atrium, caused by the decrease in intrathoracic negative pressure [31]. The decrease in hypoxemia during the nighttime could also decrease the hypoxic contraction in the pulmonary system [32]. As a result, the excretion of atrial natriuretic peptide during sleep could be reduced, resulting in a decrease in nocturia. We also presume that these surgical effects could cause decreases in urinary electrolyte excretion and nocturnal urine volume, which could reduce the frequency of nocturia.

In this research, there was a reduction from pre- to post-surgery in the BMI of all patients. After further inspection, we realized that this situation was caused by the discomfort and loss of appetite after UPPP surgery. Since all patients were being taken good care of by the medical professionals in the hospital, we did not regard this situation as critical, which was vital to the patients. We do not know the reason why surgical success is impacted by proper surgical selection or by reducing BMI after poor intake following sleep surgery. Even though the decrease in BMI reduced the AHI, we still found that the significantly decreased AHI (i.e., in the successful surgery group) resulted in a decreased frequency of nocturia. Interestingly, a similar decrease in the frequency of nocturia was also found in the unsuccessful surgery group. A possible explanation is a reduction in upper airway inflammation or a reduction in inflammatory cytokines after UPPP that results in better and more stable sleep, thus leading to a higher threshold regarding urinary urgency. In addition, the severity of OSA was also reduced by UPPP surgery, and increased airway flow was also gained even when failing to meet Sher’s criteria for surgical success, however, AHI reduction (change in AHI by UPPP) had a significant slight correlation to the score reduction of IPSS and OABSS in the current study. Therefore, the reduced disease severity might be another explanation for our findings. Debates are still ongoing as to whether decreased BMI leads to decreased nocturia or not, although there has been a report that reveals that decreased BMI does indeed lead to decreased nocturia [33]. Contrarily, other studies have revealed that a higher BMI is related to decreased nocturia [34,35].

The results of our research indicate that whether or not UPPP is successful, patients with nocturia who undergo this surgery can experience improved rates of nighttime urination. We certainly believe that more clinical research focusing on the mechanism of why unsuccessful UPPP surgery could lead to a reduction in nocturia should be conducted in the near future. In addition, further medical studies designed to determine the relationship between OSA and nighttime urination are highly encouraged [36]. This research also revealed the fact that there is a positive correlation between the AHI and the incidences of nocturia. Accordingly, patients with a higher AHI were found to have significantly higher rates of nocturia [37,38]. Therefore, more severe OSA patients, in other words, patients with higher AHI, would need to have a detailed nocturia evaluation before surgery, even though they would need to be treated for nocturia at the same time.

Although the OSA severity itself is correlated to nocturia frequency [39], we still found that UPPP contributed to a greater AHI reduction in the moderate and severe AHI groups (even if they did not reach the definition of Sher’s criteria of surgical success), that is related to significant nocturia reduction. In addition, whether surgical success occurred, whether matching Sher’s criteria or not, was related to nocturia reduction in the nocturia group. The reduction of AHI also slightly correlated to IPSS and OABSS reduction subjectively. There were some limitations in the current study. Our study was a retrospective study with non-symmetric AHI severity distribution between groups, and was performed in one single center, resulting in a relatively small sample population. Reporting bias in the questionnaires was also still inevitable. It is necessary for more prospective and controlled studies to investigate the clinical outcomes between OSA patients with nocturia after UPPP.

## 5. Conclusions

In summary, we conclude that sleep surgery is an alternative and robust treatment strategy for patients who suffer from nocturia. UPPP appears to be an effective treatment for nocturia associated with OSA, but the relationship of AHI reduction and nocturia improvement after UPPP is still unclear. However, UPPP can reduce the symptoms of OSA and can also contribute to reducing nocturia even in those who experience unsuccessful surgery. In the future, it should be determined why unsuccessful surgery also results in a decrease in the frequency of nocturia.

## Figures and Tables

**Table 1:** The characteristics of the 103 obstructive sleep apnea (OSA)-diagnosed patients.

	Group 1 Non-Nocturia Patients (*n* = 50)	Group 2 Nocturia Patients (*n* = 53)
Sex (male/female)	41/9	43/10
Age (years)	43.34 ± 10.71	42.60 ± 9.69
BMI (kg/m^2^)	26.59 ± 2.92	26.89 ± 2.64
No. of patients with nocturia		
0 episodes/night	26	0
1 episodes/night	24	0
2 episodes/night	0	37
3 episodes/night	0	12
4 episodes/night	0	4
Oxygen desaturation index	11.66 ± 8.02	27.12 ± 18.36 *
Apnea index	7.18 ± 7.67	21.56 ± 19.88 *
AHI	13.94 ± 8.22	29.99 ± 22.75 *
ESS	11.16 ± 4.29	12.54 ± 3.79
IPSS	3. 31 ± 2.83	9.52 ± 4.56 *
OABSS	1.07 ± 0.86	3.45 ± 2.34 *
Sleep architecture		
Stage N1 (%)	17.21 ± 3.56	13.45 ± 4.12
Stage N2 (%)	54.34 ± 4.55	56.56 ± 6.11
Stage N3 (%)	13.45 ± 5.37	15.52 ± 7.87
REM (%)	16.67 ± 7.56	18.77 ± 7.89
Number of PLMs per hour	7.42 ± 4.56	6.67 ± 8.32

* *p* < 0.05, significant difference between group 1 and group 2. BMI, body mass index; AHI, apnea–hypopnea index; ESS, Epworth Sleepiness Scale; IPSS, International Prostatic Symptom Score; OABSS, Overactive Bladder Symptom Score; REM, rapid eye movement; PLMs, periodic leg movements.

**Table 2:** Changes of AHI and nocturia frequency after UPPP among different OSA severity groups.

	AHI	Nocturia Frequency
Non-nocturia patients (*n* = 50)		
AHI 5~14/h (*n* = 31)	−0.28 ± 11.87	0.19 ± 1.01
AHI 15~30/h (*n* = 17)	0.53 ± 17.28	0.29 ± 1.10
AHI > 30/h (*n* = 2)	−17.90 ± 5.79	−0.50 ± 0.70
Nocturia patients (*n* = 53)		
AHI 5~14/h (*n* = 14)	5.76 ± 23.96	−1.28 ± 0.76
AHI 15~30/h (*n* = 22)	−7.68 ± 14.42 *	−1.59 ± 0.64 *
AHI > 30/h (*n* = 17)	−39.52 ± 16.32 **	−1.64 ± 0.86 **
Total patients (*n* = 103)		
AHI 5~14/h (*n* = 45)	1.59 ± 16.54	−0.26 ± 1.23
AHI 15~30/h (*n* = 39)	−4.10 ± 16.05 *	−0.76 ± 1.45 *
AHI > 30/h (*n* = 19)	−37.25 ± 16.88 **	−1.52 ± 0.90 **

* *p* < 0.05, AHI 15–30/h vs. AHI 5~14/h; ** *p* < 0.05, AHI > 30/h vs. AHI 5~14/h. UPPP, uvulopalatopharyngoplasty.

**Table 3:** Changes in BMI, nocturia frequency, AHI, and ESS in both groups for nocturia and non-nocturia patients.

Items	Group 1 Non-Nocturia Patients (*n* = 50)		Group 2 Nocturia Patients (*n* = 53)	
	Pre-Surgery	Post-Surgery	Pre-Surgery	Post-Surgery
BMI (kg/m^2^)	26.59 ± 2.92	25.49 ± 2.68 ^+^	26.89 ± 2.64	25.69 ± 3.13 ^+^
Nocturia frequency	0.48 ± 0.50	0.68 ± 0.89	2.27 ± 0.62 *	0.84 ± 0.91 ^+^
AHI	13.94 ± 8.22	13.23 ± 16.12	29.99 ± 22.75	15.64 ± 16.61 ^+,^*
ESS	11.16 ± 4.29	9.52 ± 3.79	12.54 ± 3.79	10.16 ± 4.27

* *p* < 0.05, significant difference between group 1 and group 2. ^+^ *p* < 0.05, significant difference between pre-surgery and post-surgery. BMI, body mass index; AHI, apnea–hypopnea index; ESS, Epworth Sleepiness Scale.

**Table 4:** Changes in BMI, nocturia frequency, AHI, and ESS only in non-nocturia patients (*n* = 50).

Items	Subgroup 1 Unsuccessful Surgery (*n* = 18)		Subgroup 2 Successful Surgery (*n* = 32)	
	Pre-Surgery	Post-Surgery	Pre-Surgery	Post-Surgery
BMI	26.23 ± 2.76	25.58 ± 4.58	26.85 ± 2.98	25.44 ± 6.75
Nocturia frequency	0.46 ± 0.51	0.61 ± 4.23	0.50 ± 0.77	0.70 ± 0.63
AHI	13.56 ± 15.26	14.53 ± 14.53	14.02 ± 12.36	11.67 ± 10.41 ^+,^*
ESS	11.36 ± 4.58	10.32 ± 3.87	10.78 ± 3.63	9.32 ± 5.31

* *p* < 0.05, significant difference between subgroup 1 and subgroup 2. ^+^ *p* < 0.05, significant difference between pre- and post-surgery. BMI, body mass index; AHI, apnea–hypopnea index; ESS, Epworth Sleepiness Scale.

**Table 5:** Changes in BMI, nocturia frequency, AHI, and ESS only in nocturia patients (*n* = 53).

Items	Subgroup 1 Unsuccessful Surgery (*n* = 20)		Subgroup 2 Successful Surgery (*n* = 33)	
	Pre-Surgery	Post-Surgery	Pre-Surgery	Post-Surgery
BMI	27.10 ± 2.69	25.78 ± 3.58	26.76 ± 2.64	25.64 ± 2.88 ^+^
Nocturia frequency	2.35 ± 0.58	0.59 ± 1.09 ^+^	2.39 ± 0.65	0.78 ± 0.78 ^+^
AHI	22.04 ± 15.31	26.49 ± 18.37	34.81 ± 25.27	9.07 ± 11.41 ^+,^*
ESS	12.40 ± 3.45	10.45 ± 4.31	12.63 ± 4.15	10.00 ± 4.31

* *p* < 0.05, significant difference between subgroup 1 and subgroup 2; ^+^ *p* < 0.05, significant difference between pre- and post-surgery. BMI, body mass index; AHI, apnea–hypopnea index; ESS, Epworth Sleepiness Scale.
